# Arterial stiffening in children with early chronic kidney disease is associated with blood pressure but not decline in kidney function: a longitudinal study from the HOT-KID cohort

**DOI:** 10.1007/s00467-026-07157-1

**Published:** 2026-01-26

**Authors:** Louise Keehn, Phil J. Chowienczyk, Rodney Gilbert, Andrew Lunn, Heather Maxwell, Henry Morgan, Mohan Shenoy, Rukshana Shroff, Pushpa Subramaniam, Jane Tizard, Yincent Tse, Manish D. Sinha

**Affiliations:** 1 Department of Clinical Pharmacology, British Heart Foundation Centre, King’s College London, London, UK; 2 Department of Paediatric Nephrology, Southampton General Hospital, Southampton, UK; 3 Department of Paediatric Nephrology, Nottingham University Hospital NHS Trust, Nottingham, UK; 4 Department of Paediatric Nephrology, Royal Hospital for Sick Children, Glasgow, UK; 5 Department of Paediatric Nephrology, Alder Hey Children’s Hospital, Liverpool, UK; 6 Department of Paediatric Nephrology, Royal Manchester Children’s Hospital Manchester, Manchester, UK; 7 Department of Paediatric Nephrology, UCL Great Ormond Street Hospital and Institute of Child Health, London, UK; 8 Department of Paediatrics, St Georges Hospital, London, UK; 9 Department of Paediatric Nephrology, Bristol Royal Hospital for Children, Bristol, UK; 10 Department of Paediatric Nephrology, Great North Children’s Hospital, Newcastle Upon Tyne, UK; 11 Department of Paediatric Nephrology, Evelina London Children’s Hospital, Westminster Bridge Road, London, UK; 12 Faculty of Life Sciences and Medicine, King’s College London, London, UK

**Keywords:** Arterial stiffening, CKD, Blood pressure, Children, Kidney, Pulse wave velocity

## Abstract

**Background:**

Children with chronic kidney disease (CKD) are at risk of hypertension and increased arterial stiffness. We examined the roles of blood pressure (BP) and kidney function in development of arterial stiffening in children with early CKD, compared to healthy children.

**Methods:**

Children who attended for two measurements (mean interval 3.1 ± 1.4 years) of carotid-femoral pulse wave velocity (PWV) as part of the HOT-KID study were included. Annual progression of PWV (PWV_AP_) was compared for children with CKD (*n* = 106) versus healthy controls (*n* = 45), adjusting for mean arterial pressure (MAP) and other risk factors at baseline and follow-up. Multivariable linear regression analyses identified variables significantly associated with PWV_AP_ for each group.

**Results:**

There was no significant difference in PWV_AP_ between children with CKD and those without, when adjusted for key covariates at baseline and follow-up (0.12 ± 0.03 m/s/year and 0.12 ± 0.05 m/s/year respectively, *P* = 0.977). In healthy controls, PWV_AP_ was independently associated with annual progression of MAP (MAP_AP_, *β* = 0.49, *P* = 0.006), whereas in children with CKD, PWV_AP_ was strongly associated with both baseline MAP and MAP_AP_ (*β* = 0.26, *P* = 0.007 and *β* = 0.53, *P* < 0.001, respectively) but not baseline or change in estimated glomerular filtration rate.

**Conclusions:**

These results indicate that there is no demonstrable difference in arterial stiffness between children with early CKD and those without. Renal function in early CKD does not appear to affect arterial stiffening, independent of the BP. The strong association between arterial stiffening and MAP suggests a need for careful BP control in children with CKD.

**Graphical Abstract:**

A higher resolution version of the Graphical abstract is available as Supplementary information.

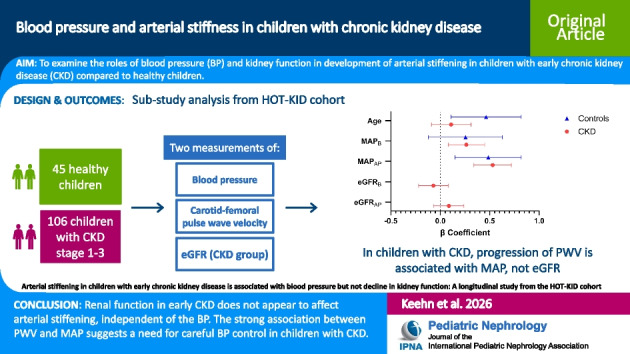

**Supplementary Information:**

The online version contains supplementary material available at 10.1007/s00467-026-07157-1.

## Introduction

Increased mortality and morbidity in children and young people (CYP) with chronic kidney disease (CKD) results from both progression of CKD and from cardiovascular disease [1–5]. Increased arterial stiffness in CKD is known to contribute to both adverse cardiac and renal (progression of CKD) outcomes in adults [6–8] and is an important predictor of premature mortality for those with CKD [7]. In CYP with CKD, increased arterial stiffness is seen in those with pre-dialysis CKD [9, 10], is worse in those on dialysis [11–13] and does not appear to reverse with transplantation [14].

Understanding the aetiology of arterial stiffening is key to its treatment. The aetiology of arterial stiffness in CYP with CKD is likely to differ from that in older people with CKD in whom age-related degenerative changes in connective tissue are seen and develop over several decades [15]. However, there are few longitudinal data examining the potential determinants of arterial “stiffening” and the progression of arterial stiffness in childhood CKD. Recent work by Azukaitis et al*.* suggested that arterial stiffening in children with moderate to severe CKD is associated more with age and blood pressure (BP), than decline in kidney function [16].

The Hypertension Optimal Treatment in Children with Chronic Kidney Disease (HOT-KID) study included an open-label, parallel group, multicentre randomised controlled trial examining effects of intensive versus less intensive BP control on cardiac remodelling that has been previously reported [17]. As part of the HOT-KID study, we also completed an observational study in children with and without CKD in which other hypertension-mediated target organ damage was examined. Here, we report the longitudinal results for arterial stiffening measured by carotid-femoral pulse wave velocity (PWV). The main objective of this exploratory analysis is to examine the association between arterial stiffening (as longitudinal change in PWV) and BP and kidney function.

## Methods

### HOT-KID study recruitment and visits

The HOT-KID study comprised a parallel, open-label, multicentre, randomised controlled trial and an observational study arm, recruiting children between 2 and 15 years with CKD stages 1–4 across 14 UK centres. Healthy children in the same age range were recruited contemporaneously from the background population [17, 18]. All children in both the randomised and observation arms of the study were invited to attend for annual visits at their recruiting hospital, where they had standardised auscultatory office BP measured and a range of cardiac and vascular measurements performed. Aortic stiffness, measured as PWV using carotid-femoral applanation tonometry, was obtained in children who were able to tolerate the procedure and in whom trained investigators were available to perform the measurement at the local study site. Inter-observer error was minimised by the investigators from the lead centre (Evelina London Children’s Hospital, King’s College London) travelling to external sites to perform all measurements.

### Sub-study participants

Data on arterial stiffness from the HOT-KID study has not been published previously. For this study, we included children with CKD and healthy controls who had at least two visits where office BP had been measured on the same day as PWV. We defined the first included vascular assessment as the ‘baseline’ visit and the last included vascular assessment as the ‘final visit’ in this study analysis.

**Fig. 1:**
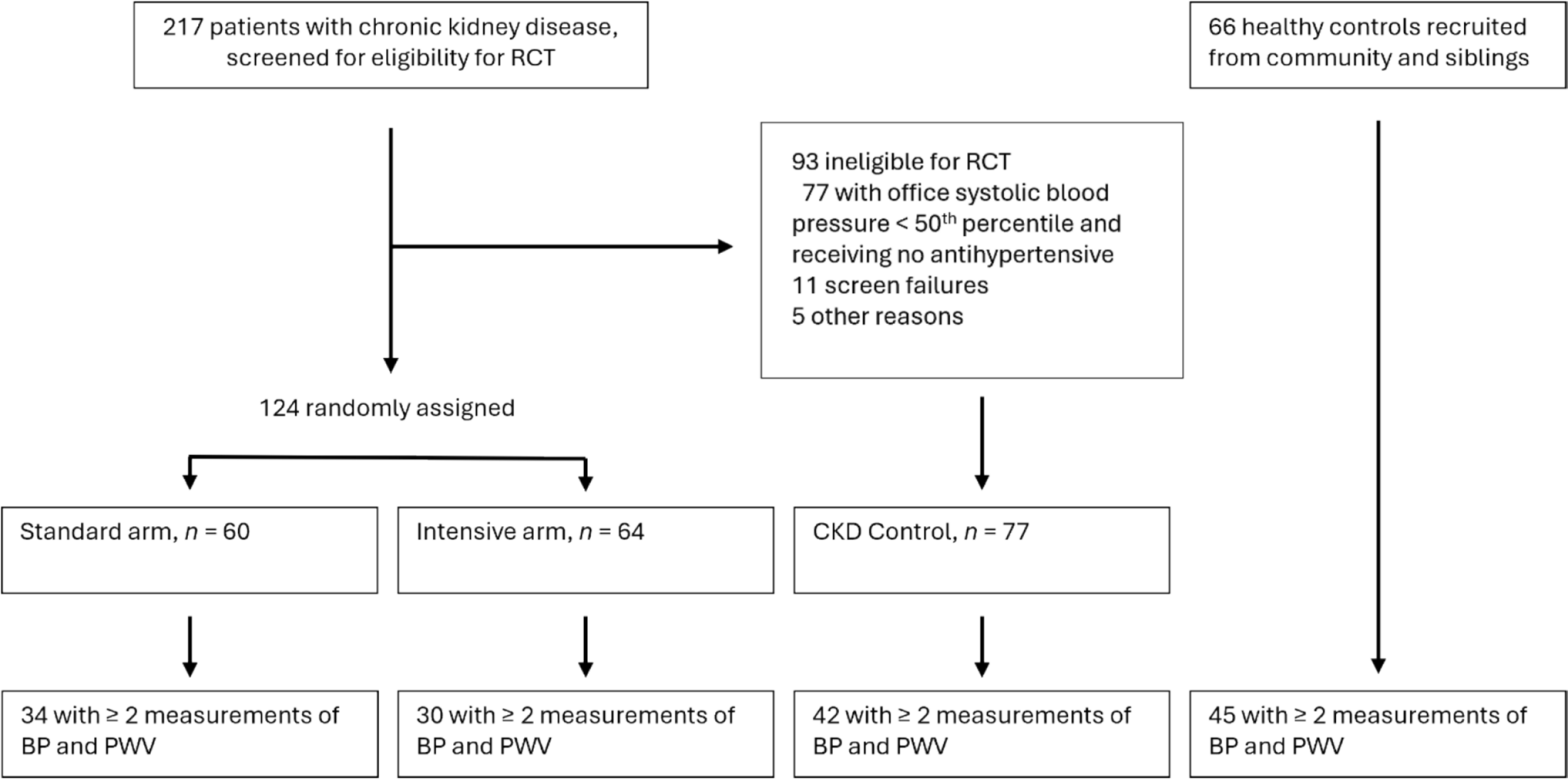
HOT-KID study numbers and participant eligibility for sub-study evaluating development of arterial stiffening

One hundred and twenty-four children with CKD participated in the HOT-KID RCT [17]. Of these 124, only 64 had multiple measurements of PWV; hence, the RCT data was insufficient to analyse the effect of longitudinal BP intervention on arterial stiffness. From the observation groups of the HOT-KID study, a further 45 healthy controls and 42 subjects with CKD with multiple measurements of PWV were entered into the analysis, as shown in Fig. 1. Overall, this study analysis included data from 10 out of the 14 HOT-KID recruiting sites.

All study procedures and investigations were carried out in accordance with the Declaration of Helsinki. The HOT-KID study protocol was approved by the UK National Research Ethics Committee (10/H0802/13), all participating institutions, and relevant regulatory authorities.

### Measurements

Baseline and subsequent visits were carried out at the subject’s local site within Clinical Research Facilities when possible. At each visit, height and weight were measured and current antihypertensive therapy documented. Ethnicity status was as self-described by the participant. Seated BP was measured following 5 min of rest by a trained health professional, using auscultation and aneroid sphygmomanometer with an appropriately sized arm cuff and inflation of the cuff to a pressure ~20–30 mmHg higher than the systolic BP. BP was measured thrice in quick succession and the average values were recorded. Blood and urine samples were obtained from all children with CKD but were optional for healthy subjects. Estimated GFR (eGFR) was calculated using the Schwartz formula and CKD staged as per existing KDIGO definitions [19, 20]. Thirty-five healthy children had blood tests on one of their visits. Clinical markers of mineral bone disease including serum calcium, phosphate, intact parathyroid hormone, and 25-hydroxy vitamin D3 concentrations and serum haemoglobin and albuminuria were measured in a subsample of CKD patients on the day of the vascular assessments. Underweight, overweight and obese categories were defined as BMI *z*-scores < –2, > 1 and > 2, respectively [21]. Short stature was defined as height > 2 SD below the mean for age and sex.

The SphygmoCor system (AtCor, Australia) was used to measure sequential ECG-referenced carotid-femoral PWV. The path length, from the suprasternal notch to the femoral pulse at the point of applanation, was measured using a tape measure, this being the methodology standardised in our laboratory before the guidance advocating use of a slightly different path length measurement (carotid to femoral) [22]. PWV measurements were performed supine after a period of rest, and three measurements were taken, with the average used for analysis. All measurements were performed by trained observers who travelled across all sites with the same device and had completed in-house training and assessment to ensure inter-observer coefficient of variation was < 10%.

### Data analysis

Analysis was performed using SPSS 28 (IBM, Chicago, Illinois, USA). Differences in baseline characteristics (subscript ‘B’) between subjects with CKD and healthy controls were analysed by independent sample Student’s *t*-test for continuous variables and Chi-squared test for categorical variables. Univariate and multivariate linear regression was used to examine associations between PWV and BP and other risk factors. All variables were standardised before entering into linear regression models; therefore, standardised regression coefficients are presented, with corresponding 95% confidence intervals. Mean arterial BP (MAP) was used to represent BP as the primary analysis to avoid collinearity between other measurements of BP and because pulse pressure is thought to be determined in large part by PWV. Additional versions using SBP and DBP instead of MAP are shown in the supplementary Online Resource. For the longitudinal analysis, annual progression (subscript ‘AP’) of PWV, MAP, heart rate (HR), body mass index (BMI), weight and eGFR was calculated by dividing the absolute difference in each measurement from follow-up to baseline by the years between baseline and follow-up. General linear models were used to calculate estimated marginal means (with confidence intervals) of rate of progression of PWV. Sub-group analysis was performed in participants with biochemical and haematological markers of CKD-associated mineral bone disease and anaemia.

As a secondary analysis, PWV *z*-scores were calculated from height and sex by reference to a standardised population [23]. However, 29% of controls and 25% of children with CKD had a PWVz measurement above the 95th percentile as defined by the reference dataset, indicating that the cohort and the reference dataset were not comparable, see Online Resource Table S1. Hence, all analysis was performed using raw values of PWV and adjusting for pertinent covariates such as age and sex.

This article follows the STROBE (Strengthening the Reporting of Observational Studies in Epidemiology) [24] reporting guidelines.

## Results

This sub-study included 151 children (46% female), aged 3–10 years, of which 45 (30%) were healthy controls, and 41 (27%) had CKD Stage 1, 36 (24%) had CKD Stage 2 and 29 (19%) had CKD Stage 3. No children with CKD 4 had longitudinal measurements of PWV, and therefore, there were none eligible for inclusion in this sub-study. The primary cause of chronic kidney disease in 65 of 106 children (61%) was congenital anomalies of the kidney and urinary tract, and thus, CKD was lifelong in these children. At baseline, the average eGFR was 85 ml/min/1.73 m^2^ in those with CKD. Of the subjects with CKD, 40% were taking antihypertensive medication on their baseline visit (mainly angiotensin converting enzyme (ACE) inhibitors). There was no significant difference at baseline in demographic characteristics and vascular measurements including age, sex, ethnicity or PWV between children with CKD and those without (Table 1). There were no differences between healthy controls and children with CKD stages 1, 2 and 3 for baseline PWV (Table 1).

**Table 1:** Subject characteristics at baseline

*n*	Controls	CKD Stage 1	CKD Stage 2	CKD Stage 3	All CKD	Overall*** P***
	45	41	36	29	106	
Age (years)	10.7 ± 3.2	10.5 ± 2.9	9.9 ± 3.5	11.0 ± 3.5	10.5 ± 3.3	0.614
Sex, male (%)	19 (42)	21 (51)	23 (65)	18 (62)	62 (59)	0.187
Ethnicity						0.098
South East Asian (%)	2 (4)	2 (5)	3 (8)	2 (7)	7 (7)	
Black (%)	2 (4)	7 (17)	0	0	7 (7)	
White (%)	38 (84)	31 (76)	30 (83)	25 (86)	86 (81)	
Other (%)	3 (7)	1 (2)	3 (8)	2 (7)	6 (6)	
Height (cm)	142 ± 18	143 ± 16	137 ± 22	140 ± 20	140 ± 20	0.554
Height *z*-score	0.28 ± 1.04	0.35 ± 1.12	−0.13 ± 1.21	−0.49 ± 0.88	−0.04 ± 1.13	**0.005**^**a**^
Short stature (%)	1 (2)	0	2 (6)	3 (10)	5 (5)	0.146
Weight (kg)	40 ± 17	39 ± 16	37 ± 18	39 ± 17	38 ± 17	0.905
Weight *z*-score	0.47 ± 1.09	0.46 ± 1.29	0.22 ± 1.19	−0.01 ± 1.14	0.25 ± 1.22	0.292
Underweight	1 (2)	1 (2)	1 (3)	0	2 (2)	0.859
Overweight	9 (20)	9 (22)	6 (17)	8 (28)	23 (22)	0.752
Obese	4 (25)	6 (15)	5 (14)	1 (3)	16 (11)	0.420
BMI (kg/m^2^)	19 ± 4	19 ± 4	19 ± 4	19 ± 3	19 ± 4	0.997
BMI *z*-score	0.40 ± 1.10	0.34 ± 1.36	0.36 ± 1.31	0.36 ± 1.06	0.35 ± 1.25	0.997
Systolic BP (mmHg)	101 ± 13	101 ± 11	105 ± 10	99 ± 9	102 ± 11	0.120
Systolic BP *z*-score	−0.11 ± 1.00	−0.10 ± 1.00	0.34 ± 0.97	−0.34 ± 0.77	−0.02 ± 0.97	**0.029**^**b**^
Diastolic BP (mmHg)	59 ± 10	59 ± 13	63 ± 13	56 ± 10	59 ± 13	0.139
Diastolic BP *z*-score	−0.39 ± 1.01	−0.43 ± 1.30	−0.02 ± 1.29	−0.74 ± 1.14	−0.37 ± 1.28	0.113
MAP (mmHg)	73 ± 9	73 ± 11	77 ± 11	70 ± 8	74 ± 11	0.065
Heart rate (bpm)	81 ± 11	78 ± 12	85 ± 15	79 ± 13	81 ± 14	0.072
PWV (m/s)	5.4 ± 0.7	5.3 ± 0.8	5.6 ± 0.9	5.2 ± 0.8	5.4 ± 0.8	0.151
Antihypertensives, *n* (%)	-	13 (32)	19 (53)	10 (35)	42 (40)	0.135^c^
eGFR* (ml/min/1.73 m^2^/year)	126 ± 20	116 ± 17	76 ± 8	50 ± 8	85 ± 30	** < 0.001**^**d**^

Values are mean ± SD or *n* (%). Overall significance is presented for controls vs. all CKD groups unless otherwise stated. *P* < 0.05 highlighted in bold

*BMI* body mass index, *BP* blood pressure, *eGFR* estimated glomerular filtration rate, *MAP* mean arterial pressure, *PWV* pulse wave velocity

^*^e-GFR was measured on one of the study visits for control children (*n* = 35) but measured on the same day as baseline PWV for children with CKD

^a^*P* < 0.05 between controls vs. CKD Stage 3, and CKD Stage 1 vs. CKD Stage 3

^b^*P* < 0.05 between CKD Stage 2 and CKD Stage 3

^c^Comparison of CKD groups only

^d^*P* < 0.05 for controls vs. all CKD Stages, and between each CKD Stage (1 vs. 2, 1 vs. 3 and 2 vs. 3)

### Cross-sectional associations of arterial stiffness with demographics, blood pressure and other risk factors

Associations between PWV and demographics, BP and other risk factors in healthy children and children with CKD measured at baseline are shown in Table 2. In univariate analysis, healthy children demonstrated significant positive associations between PWV and age, BMI, BMIz, height, weight, weightz, MAP and SBP. In multivariable analysis, only age and BMIz remained significantly associated with PWV. In children with CKD, there were significant univariate associations between PWV and age, sex, height, weight, MAP and SBP. However, only age remained significantly associated with PWV in multivariable analysis. PWV was not associated with eGFR in univariate or multivariable analysis. Additional analyses including SBP or DBP instead of MAP are shown in the Online Resource Tables S2 and S3.

**Table 2:** Cross-sectional associations with PWV at study baseline, using univariate and multivariable linear regression analyses

	Controls						CKD					
	Univariate			Multivariable			Univariate			Multivariable		
	*β*	95% CI	*P*	*β*	95% CI	*P*	*β*	95% CI	*P*	*β*	95% CI	*P*
Age (years)	0.57	0.32–0.83	**< 0.001**	0.54	0.28–0.81	**<0.001**	0.31	0.12–0.49	**0.001**	0.31	0.11–0.52	**0.004**
Sex, male (%)	−0.15	−0.45–0.16	0.329	0.01	−0.22–0.24	0.934	−0.25	−0.44–−0.06	**0.009**	−0.19	−0.39–0.00	0.053
Ethnicity (non-White)	−0.17	−0.47–0.14	0.277	0.02	−0.20–0.25	0.834	0.11	−0.07–0.25	0.283	0.13	−0.06–0.31	0.189
BMI (kg/m^2^)	0.69	0.46–0.91	**< 0.001**	-	-	**-**	0.18	−0.01–0.37	0.061	-	-	-
BMIz	0.51	0.24–0.77	**< 0.001**	0.44	0.20–0.69	**<0.001**	−0.18	−0.21–0.18	0.858	−0.08	−0.27–0.10	0.379
Height (cm)	0.58	0.33–0.83	**< 0.001**	-	-	-	0.33	0.15–0.52	**< 0.001**	-	-	-
Heightz	0.23	−0.07–0.53	0.125	-	-	-	0.18	−0.01–0.37	0.061	-	-	-
Weight (kg)	0.72	0.51–0.93	**< 0.001**	-	-	**-**	0.30	0.11–0.48	**0.002**	-	-	-
Weightz	0.51	0.25–0.78	**< 0.001**	-	-	-	0.10	−0.10–0.29	0.334	-	-	-
MAP (mmHg)	0.36	0.08–0.65	**0.014**	0.04	−0.21–0.29	0.733	0.22	0.03–0.41	**0.025**	0.13	−0.06–0.32	0.165
SBP (mmHg)	0.47	0.19–0.74	**0.001**	-	-	-	0.29	0.10–0.47	**0.003**	-	-	-
DBP (mmHg)	0.20	−0.10–0.50	0.187	-	-	-	0.16	−0.04–0.35	0.111	-	-	-
HR (bpm)	0.01	−0.30–0.31	0.974	0.05	−0.20–0.31	0.681	−0.02	−0.21–0.72	0.853	0.12	−0.09–0.33	0.265
Antihypertensives (y/n)	-	-	-	-	-	-	0.00	−0.19–0.20	0.985	−0.04	−0.23–0.15	0.700
eGFR (ml/min/1.73 m^2^/year)	-	-	-	-	-	-	−0.03	−0.22–0.17	0.792	−0.04	−0.23–0.15	0.672

Multivariable analysis was performed using the enter method. *β* = standardised regression coefficient. *P* < 0.05 highlighted in bold

*BMI* body mass index, *BMIz* body mass index *z*-score, *eGFR* estimated glomerular filtration rate, *HR* heart rate,* MAP* mean arterial pressure, *SBP* systolic blood pressure, *DBP* diastolic blood pressure, *PWV* pulse wave velocity, *CI* confidence interval

There was no significant difference in baseline PWV between healthy control girls and boys (5.46 ± 0.65 m/s and 5.26 ± 0.76 m/s respectively, *P* = 0.329). The difference remained insignificant after adjustment for age, ethnicity, MAP, HR and BMIz *(P* = 0.575). In children with CKD, baseline PWV was higher in girls compared to boys (5.62 ± 0.86 m/s versus 5.19 ± 0.79 m/s, *P* = 0.009). However, the significance was reduced when adjusted for age, ethnicity, MAP, HR and BMIz (*P* = 0.052).

Subgroup analyses in those with available markers of CKD associated mineral bone disease and anaemia showed significant association of PWV with parathyroid hormone (PTH) in univariate but not in multivariable analysis, see Online Resource Table S4.

### Longitudinal associations between arterial stiffness, blood pressure and subject demographics

The mean time between baseline and final visit was 3.1 ± 1.4 years. Over the follow-up period, absolute height, weight, BMI, SBP and DBP increased significantly for healthy children and for those with CKD. BMIz, SBPz and DBPz did not significantly change over follow-up for either group (all *P* > 0.05). PWV increased by 0.26 ± 0.8 m/s for control subjects (*P* = 0.030) and 0.32 ± 0.89 m/s for CKD subjects, (*P* < 0.001); however, the difference in absolute PWV between the two groups was not significant (*P* = 0.723). There were no significant differences in the annual gains in height or weight between healthy children and controls (*P* = 0.121 and *P* = 0.935 respectively). In those with CKD, eGFR declined from 83 ± 29 ml/min/1.73 m^2^ to 75 ± 30 ml/min/1.73 m^2^ (*P* < 0.001). The proportion of subjects with CKD on antihypertensive medication did not significantly change (40% at baseline, compared to 43% at follow-up, *P* = 0.388).

There was no significant difference in the unadjusted annual rate of progression of PWV (PWV_AP_) between healthy controls and subjects with CKD (0.13 ± 0.38 m/s/year and 0.11 ± 0.44 m/s/year, respectively (*P* = 0.751). When adjusted for sex, ethnicity, baseline age (age_B_), BMIz (BMIz_B_), HR (HR_B_), MAP (MAP_B_), PWV (PWV_B_) and annual progression of BMIz (BMIz_AP_), MAP (MAP_AP_), HR (HR_AP_) and use of antihypertensives at baseline and follow-up, there was no significant difference in PWV_AP_ between healthy children and those with CKD (0.12 ± 0.05 m/s/year and 0.12 ± 0.03 m/s/year respectively, *P* = 0.977).

**Fig. 2:**
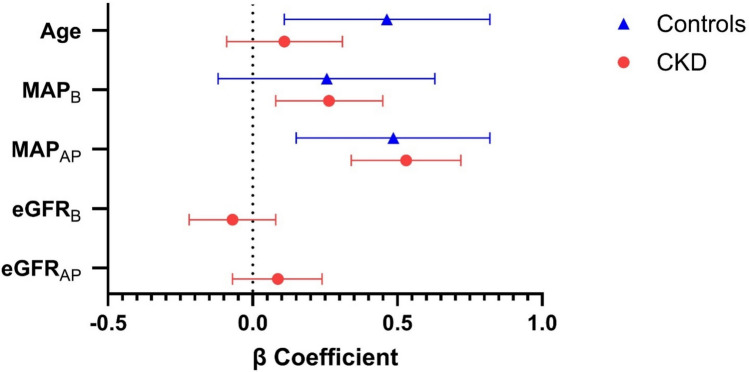
Forest plot of linear regression model examining the associations of annual progression in PWV to baseline and progression of risk factors in children with CKD and healthy controls. *β* is the standardised beta coefficient, bars represent 95% confidence intervals. eGFR_B_, estimated glomerular filtration rate at baseline; eGFR_AP_, annual progression of estimated glomerular filtration rate; MAP_B_, mean arterial pressure at baseline; MAP_AP_, annual progression in mean arterial pressure; PWV, pulse wave velocity

When the associations between progression of PWV (PWV_AP_) and baseline and follow-up measures are considered in multivariable regression analysis, age_B_, PWV_B_ and MAP_AP_ were significantly associated with PWV_AP_ in healthy control children; see Table 3 and Fig. 2. In these children, MAP_B_ was not significantly associated with PWV_AP_. In children with CKD, PWV_AP_ was not significantly associated with age_B_ but was associated with MAP_B_, PWV_B_, BMIz_B_ and MAP_AP_ (see Fig. 2). There were no significant associations between use of antihypertensives at the baseline or follow-up visit and PWV_AP_ in children with CKD. eGFR at baseline and follow-up was also not significantly associated with PWV_AP_ in subjects with CKD.

**Table 3:** Longitudinal associations between progression of PWV and baseline and follow-up risk factors, using multivariable linear regression analyses

	Controls			CKD		
	*β*	95% CI	*P*	*β*	95% CI	*P*
**Baseline factors**						
Age_B_ (years)	0.46	0.11–0.82	**0.012**	0.11	−0.09–0.31	0.285
Sex	0.04	−0.21–0.29	0.741	−0.08	−0.24–0.09	0.374
Ethnicity (non-White)	−0.14	−0.41–0.12	0.279	0.04	−0.12–0.19	0.633
BMIz_B_	−0.23	−0.54–0.07	0.132	−0.17	−0.33– −0.02	**0.031**
MAP_B_ (mmHg)	0.26	−0.12–0.63	0.175	0.26	0.08–0.45	**0.007**
HR_B_ (bpm)	0.27	−0.02–0.56	0.064	0.02	−0.16–0.21	0.798
PWV_B_ (m/s)	−0.63	−0.98– −0.28	**< 0.001**	−0.59	−0.76– −0.42	**<0.001**
Antihypertensives (y/n)	-	-	-	0.15	−0.10–0.39	0.237
eGFR_B_ (ml/min/1.73 m^2^/year)	-	-	-	−0.07	−0.22–0.08	0.380
**Follow-up measures**						
BMIz_AP_	0.05	−0.21–0.32	0.695	−0.09	−0.25–0.07	0.280
MAP_AP_ (mmHg)	0.49	0.15–0.82	**0.006**	0.53	0.34–0.72	**< 0.001**
HR_AP_ (bpm)	0.26	−0.01–0.53	0.060	0.05	−0.13–0.22	0.603
Antihypertensives (y/n)	-	-	-	−0.19	−0.44–0.06	0.139
eGFR_AP_ (ml/min/1.73 m^2^/year)	-	-	-	0.09	−0.07–0.24	0.265

Multivariable analysis was performed using the enter method. *β* = standardised regression coefficient. *P* <0.05 highlighted in bold

*BMIz* body mass index *z*-score at baseline, *BMIz*_AP_ annualprogression in body mass index z-score, *eGFR*_B_ estimated glomerular filtration rate at baseline, *eGFR*_AP_ annual progression in estimated glomerular filtration rate, *HR*_B_ heart rate at baseline, *HR*_AP_ annual progression in heart rate, *MAP*_B_ mean arterial pressure at baseline, *MAP*_AP_ annualprogression in mean arterial pressure, *PWV* pulse wave velocity, *CI* confidence interval

Using multivariable regression models with SBP or DBP instead of MAP, we observed no change in the original findings in those with CKD (Online Resource Tables S5 and S6). There was no significant difference in PWV_AP_ between healthy girls and boys (0.14 ± 0.39 m/s/year and 0.12 ± 0.38 m/s/year, *P* = 0.877). When adjusted for age, ethnicity, MAP, HR and BMIz at baseline and follow-up, there was still no difference in PWV_AP_ between healthy girls and boys (*P* = 0.926). For children with CKD, there was no significant difference in the PWV_AP_ between girls and boys (0.08 ± 0.33 m/s/year and 0.13 ± 0.50 m/s/year, *P* = 0.877). When adjusted for age, ethnicity, MAP, HR and BMIz at baseline and follow-up, no significant difference in PWV_AP_ between girls and boys with CKD was observed (*P* = 0.568).

## Discussion

The main findings of this study are threefold: first, that during childhood in those with early CKD arterial stiffness is comparable with healthy peers; second, that progression of arterial stiffness (as measured by aortic PWV) is closely associated with MAP; third, that renal function does not appear to be associated with arterial stiffness independently of the BP during early CKD. Overall, this study provides initial data to improve current understanding regarding the progression of CKD and its relationship with increasing arterial stiffness.

Our findings expand on our previous cross-sectional work which demonstrated that arterial stiffness was comparable between age- and sex-matched healthy children and those with CKD, with differences in elasticity driven by BP and not renal function [9]. Savant et al*.* also found that arterial stiffness was comparable between normal children and those with mild CKD and that GFR had no significant association with PWV, although both studies were limited in their cross-sectional designs [9, 10]. A recent longitudinal analysis by Azukaitis et al*.* found that higher PWV was associated with MAP over several years of follow-up but not eGFR [16]. Their analysis studied subjects with mean baseline eGFR 27 ml/min/1.73 m^2^, significantly lower than our study [16]. Considering this study alongside our own suggests that arterial stiffness is less likely to be directly affected by renal function within any stage of pre-dialysis CKD although collectively the two studies include < 500 children with pre-dialysis CKD, highlighting the relatively few data to date. The relevance of understanding arterial stiffness in CKD is highlighted by data that demonstrate stiffening to be a predictor of premature mortality in adults with CKD [7].

By including a control group in this longitudinal analysis, we have been able to compare associations with PWV and evaluate their development in those with CKD compared to healthy vascular aging. The strong associations with age and PWV in univariate analyses are in keeping with normal physiological changes [23]. In longitudinal analyses, age remains significantly associated with PWV for healthy controls but not subjects with CKD, who instead demonstrate significant association with baseline MAP. These data suggest that even in early CKD, BP becomes pertinent for arterial stiffening, beyond the normal increment seen with healthy growth. Both healthy controls and children with CKD demonstrated an association between progression in MAP and progression in PWV. This could reflect structural change, or is simply a product of the contemporaneous distending pressure at the time of PWV measurement [25, 26].

Increased arterial stiffness seen in patients with advanced CKD is due in part to the deposition of minerals and calcification of the medial layer of the arterial wall [27]. In our study of children with pre-dialysis CKD, we did not see any significant associations between PWV and secondary hyperparathyroidism when adjusted for other factors, which is in keeping with other studies in children with pre-dialysis CKD [16]. It is possible that markers of CKD-associated bone mineral disease may be more associated with PWV when children reach more severe stages of CKD including kidney failure [13, 28]. This is supported by a recent study showing that PWV increases in children with CKD in the 2 years prior to kidney replacement therapy in parallel with increases in biochemical markers of kidney disease such as phosphate and PTH [29].

Elevated PWV is also seen in children without CKD, who have primary hypertension [30]. In these subjects, it is likely that BP is the main determinant of the increase in PWV [31]. This is broadly in keeping with the results from this study, which shows that in children at risk of increased arterial stiffening (those with CKD), there was a greater association seen with BP than in healthy controls. However, the lack of difference in PWV between subjects with the most advanced CKD in our study (Stage 3) and healthy controls suggests that in these early stages of CKD, significant structural change from CKD itself is not yet apparent. In cohorts such as ours, comprising young subjects with early CKD and well controlled hypertension, PWV may not provide any additional prognostic cardiovascular risk stratification. A future focus for research would be to identify children with CKD who are likely to be at increased risk of arterial stiffening through other exacerbating comorbidities and metabolic derangements or in those who are unable to achieve satisfactory BP control over a longer duration.

In adults, BP and PWV are closely linked by a bidirectional relationship [26, 32]. It is possible that such a relationship may also exist between arterial stiffness and PWV during the later stages of CKD [33]. Interruption of the cycles of progression between BP, PWV and CKD at an early stage in life and in the disease process is therefore likely to be most impactful in the prevention of progression of arterial stiffening in children with CKD, and in turn its cardiovascular sequelae. Future advances may aim to reverse pathological changes in arterial stiffening directly, but currently, there is no therapeutic mechanism to achieve this [34]. As such, it remains advisable to maintain careful control of BP in children with CKD through lifestyle management and use of antihypertensive therapy. As per the findings from the HOT-KID study, titration of antihypertensive therapy to achieve SBP at the 50th percentile represents an optimal target for prevention of increased left ventricular mass [17]. Due to the close association of BP and PWV, such a reduction in BP would also likely decrease the contemporaneous PWV, although this remains to be tested in larger trials.

There are some important limitations to our study. Firstly, the number of children in the study, particularly controls, was quite small. However, despite this, numbers were sufficient to demonstrate strong associations between the key variables of BP, PWV and age although we cannot rule out that milder associations may have been detected with greater numbers in the control group in particular. The longitudinal nature of the analysis in controls and in children with mild CKD makes this analysis novel despite the small numbers. It is worth noting that none of the children in our study had uncontrolled hypertension; therefore, the range of BP evaluated here is somewhat narrow. Forty percent of the subjects with CKD were on antihypertensive therapy at baseline. Whilst this proportion did not significantly change over the course of follow-up, we were unable to adjust for varying doses or different types of medication. It is possible that some forms of antihypertensive therapies, particularly angiotensin-converting enzyme inhibitors, may exert beneficial effects on arterial stiffening independent of the BP [35]*,* which could have slowed the process of arterial stiffening in those children on this type of therapy. Our results are only generalisable to children with early stages of CKD, and who were no different to healthy controls in terms of growth, weight distribution or BP at the start at study baseline. However, this group’s comparability to the healthy controls at study baseline does make them an ideal group in which to investigate the origins of arterial stiffness, before there are evident CKD-driven changes to BP and body morphology. Our findings are also in keeping with other studies which have examined arterial stiffness at other stages of CKD [10, 16].

## Conclusion

Arterial stiffness and its progression are comparable between children with early CKD and those without. Concurrent MAP is strongly associated with progression of PWV in healthy children and those with CKD. However, arterial stiffening in children with CKD, in contrast to healthy controls, is also associated with baseline MAP, possibly indicating a greater susceptibility to pressure-mediated changes in arterial structure by the abnormal biochemical processes inherent in CKD. In children with mild CKD, renal function as measured by eGFR does not appear to be associated with arterial stiffness independently of the blood pressure.

## Supplementary Information

Below is the link to the electronic supplementary material.

ESM 1 (39.1 KB DOCX)

ESM 2 Graphical abstract (264 KB PPTX)

## Data Availability

The data supporting this study are available from the corresponding author on reasonable request.
