# Supplementary material for: Arterial stiffening in children with early chronic kidney disease is associated with blood pressure but not decline in kidney function: a longitudinal study from the HOT-KID cohort
Source: Pediatr Nephrol. 2026 Jan 26;41(7):2099–108. doi: 10.1007/s00467-026-07157-1 (PMC13197352; doi:10.1007/s00467-026-07157-1)
Supplement: Supplementary file 1 — (39.1 KB DOCX) [file 467_2026_7157_MOESM1_ESM.docx]

**SUPPLEMENTAL MATERIAL**

**arterial stiffening in children with early chronic kidney disease is associated with blood pressure but not DECLINE IN kidney function: A LONGITUDINAL STUDY from the HOT-KID COHORT**

**Pediatric Nephrology**

**Short Title: Blood Pressure and Arterial Stiffness in Children with Chronic Kidney Disease**

Louise Keehn^a^, MSc; Phil Chowienczyk^a^, FRCP; Rodney Gilbert^b^, FRCPCH; Andrew Lunn^c^ , BM, Heather Maxwell^d^, MD; Henry Morgan^e^ MBChB, Mohan Shenoy^f^, MRCPCH ; Rukshana Shroff^g^, PhD; Pushpa Subramaniam^h^, FRCPCH; Jane Tizard^i^, MBBS; Yincent Tse^j^, MBChB; Manish D. Sinha^k^ , PhD

^a^ Department of Vascular Risk and Surgery, King’s College London British Heart Foundation Centre, St Thomas’ Hospital, London, UK

^b^ Department of Paediatric Nephrology, Southampton General Hospital, Southampton, UK.

^c^ Department of Paediatric Nephrology, Nottingham University Hospital NHS Trust, Nottingham, UK

^d^ Department of Paediatric Nephrology, Royal Hospital for Sick Children, Glasgow, UK

^e^ Department of Paediatric Nephrology, Alder Hey Children’s Hospital, Liverpool, UK

^f^ Department of Paediatric Nephrology, Royal Manchester Children’s Hospital Manchester, UK

^g^ Department of Paediatric Nephrology, UCL Great Ormond Street Hospital and Institute of Child Health, London, UK

^h^ Department of Paediatrics, St Georges Hospital, London, UK

^i^ Department of Paediatric Nephrology, Bristol Royal Hospital for Children, Bristol, UK

^j^ Department of Paediatric Nephrology, Great North Children’s Hospital, Newcastle upon Tyne, UK

^k^ Department of Paediatric Nephrology, Evelina London Children’s Hospital, Westminster Bridge Road, London, UK.

^l^ King’s College London British Heart Foundation Centre, St Thomas’ Hospital, London, UK

**Corresponding author:** Professor Manish D. Sinha, Department of Paediatric Nephrology, Evelina London Children’s Hospital, Westminster Bridge Road, London, UK. Manish.sinha@nhs.net

**Table S1: PWV and PWV *z*-scores for control and CKD subjects**

|  | **Controls** | | **CKD** | |  |  |
| --- | --- | --- | --- | --- | --- | --- |
|  | **Mean ± SD** | **Range** | **Mean ± SD** | **Range** | ***P*** | |
| PWV (m/s) | 5.4 ± 0.7 | 4.0 – 7.0 | 5.4 ± 0.8 | 3.1 – 7.8 | 0.987 | |
| PWV *z*-score | 0.96 ± 0.84 | -1.04 – 2.69 | 0.98 ± 1.22 | -2.65 – 5.48 | 0.914 | |
| PWVz > 1.645, n (%) | 13 (29) | - | 26 (25) | - | 0.356 | |

PWV: pulse wave velocity

**Table S2: Cross-sectional associations with PWV at study baseline with SBP in the multivariable model instead of MAP.**

|  | **Control** | | | **CKD** | | | |  |
| --- | --- | --- | --- | --- | --- | --- | --- | --- |
|  | **Multivariable** | | | **Multivariable** | | | |  |
|  | **β** | **95% CI** | ***P*** | **β** | **95% CI** | ***P*** | |  |
| Age (years) | 0.54 | 0.28 – 0.81 | **<0.001** | 0.29 | 0.08 – 0.50 | | **0.008** | |
| Sex, male (%) | 0.01 | -0.22 – 0.24 | 0.934 | -0.17 | -0.37 – 0.02 | | 0.086 | |
| Ethnicity (non- white) | 0.02 | -0.20 – 0.25 | 0.834 | 0.13 | -0.06 - 0.31 | | 0.187 | |
| BMIz | 0.44 | 0.20 – 0.69 | **<0.001** | -0.09 | -0.27 – 0.10 | | 0.335 | |
| SBP (mmHg) | 0.04 | -0.21 – 0.29 | 0.733 | 0.18 | -0.20 - 0.38 | | 0.077 | |
| HR (bpm) | 0.05 | -0.20 – 0.31 | 0.681 | 0.10 | -0.11 – 0.31 | | 0.347 | |
| Antihypertensives (y/n) | - | - | - | -0.05 | -0.24 – 0.14 | | 0.607 | |
| eGFR (ml/min/1.73m^2^/year) | - | - | - | -0.03 | -0.22 – 0.15 | | 0.732 | |

Multivariable analysis was performed using the enter method. β = standardised regression coefficient. BMI: body mass index; BMIz: body mass index *z*-score, eGFR: estimated glomerular filtration rate; HR: heart rate; MAP_:_ mean arterial pressure; SBP: systolic blood pressure, PWV: pulse wave velocity; CI, confidence interval. *P* <0.05 highlighted in bold.

**Table S3: Cross-sectional associations with PWV at study baseline with DBP in the multivariable model instead of MAP.**

|  | **Control** | | | **CKD** | | | |  |
| --- | --- | --- | --- | --- | --- | --- | --- | --- |
|  | **Multivariable** | | | **Multivariable** | | | |  |
|  | **β** | **95% CI** | ***P*** | **β** | **95% CI** | ***P*** | |  |
| Age (years) | 0.56 | 0.31 – 0.80 | **<0.001** | 0.33 | 0.12 – 0.53 | | **0.003** | |
| Sex, male (%) | 0.01 | -0.23 – 0.24 | 0.945 | -0.20 | -0.40 – -0.01 | | **0.040** | |
| Ethnicity (non- white) | 0.02 | -0.21 – 0.25 | 0.853 | 0.13 | -0.06 - 0.32 | | 0.176 | |
| BMIz | 0.45 | 0.21 – 0.69 | **<0.001** | -0.08 | -0.27 – 0.11 | | 0.411 | |
| DBP (mmHg) | 0.01 | -0.22 – 0.25 | 0.902 | 0.10 | -0.09 - 0.29 | | 0.299 | |
| HR (bpm) | 0.05 | -0.20 – 0.31 | 0.681 | 0.12 | -0.09 – 0.33 | | 0.250 | |
| Antihypertensives (y/n) | - | - | - | -0.03 | -0.22 – 0.16 | | 0.734 | |
| eGFR (ml/min/1.73m^2^/year) | - | - | - | -0.04 | -0.23 – 0.15 | | 0.659 | |

Multivariable analysis was performed using the enter method. β = standardised regression coefficient. BMI: body mass index; BMIz: body mass index *z*-score, eGFR: estimated glomerular filtration rate; HR: heart rate; MAP_:_ mean arterial pressure; DBP: diastolic blood pressure, PWV: pulse wave velocity; CI, confidence interval. *P* <0.05 highlighted in bold.

**Table S4: Univariate associations of clinical, demographic and biochemical variables with baseline PWV and multivariable linear regression analysis in subgroup of children with available blood tests on the day of measurement of arterial stiffness.**

|  | **Univariate** | | | | **Multivariable with PTH**  ***N* = 80** | | | |  |
| --- | --- | --- | --- | --- | --- | --- | --- | --- | --- |
|  | ***N*** | **β** | **95% CI** | ***P*** | | **β** | **95% CI** | ***P*** | |
| Age (years) | 106 | 0.31 | 0.12 – 0.49 | **0.001** | | 0.33 | 0.10 – 0.57 | **0.006** | |
| Sex, male (%) | 106 | -0.25 | -0.44- -0.06 | **0.009** | | -0.24 | -0.46 - -0.02 | **0.033** | |
| Ethnicity (non- white) | 106 | 0.11 | -0.07 – 0.25 | 0.283 | | 0.08 | -0.13 – 0.29 | 0.458 | |
| BMIz | 106 | -0.18 | -0.21 – 0.18 | 0.858 | | -0.06 | -0.27 – 0.15 | 0.549 | |
| MAP (mmHg) | 106 | 0.22 | 0.03 – 0.41 | **0.025** | | 0.11 | -0.10 – 0.32 | 0.294 | |
| HR (bpm) | 106 | -0.02 | -0.21 – 0.72 | 0.853 | | 0.10 | -0.13 – 0.33 | 0.387 | |
| Antihypertensives (y/n) | 106 | 0.00 | -0.19 – 0.20 | 0.985 | | -0.01 | -0.23 – 0.21 | 0.923 | |
| eGFR (ml/min/1.73m^2^/year) | 106 | -0.03 | -0.22 – 0.17 | 0.792 | | 0.08 | -0.14 – 0.29 | 0.480 | |
| Haemoglobin (g/L) | 95 | 0.03 | -0.18 – 0.23 | 0.810 | | - | - | - | |
| Serum calcium (mmol/l) | 93 | -0.08 | -0.29 – 0.13 | 0.471 | | - | - | - | |
| Phosphate (mmol/l) | 95 | -0.07 | -0.28 – 0.14 | 0.507 | | - | - | - | |
| Albumin (g/L) | 96 | 0.08 | -0.13 – 0.28 | 0.456 | | - | - | - | |
| Log UACR | 48 | 0.07 | -0.24 – 0.38 | 0.656 | | - | - | - | |
| Log PTH | 80 | 0.29 | 0.08 – 0.52 | **0.008** | | 0.18 | -0.03- 0.39 | 0.090 | |
| Log 25-hydroxy vitamin D3 | 50 | -0.05 | -0.37 – 0.26 | 0.743 | | - | - |  | |

Multivariable analysis was performed using the enter method. β = standardised regression coefficient. BMIz : body mass index *z*-score; eGFR: estimated glomerular filtration rate; HR: heart rate; MAP_:_ mean arterial pressure; PTH: parathyroid hormone; PWV: pulse wave velocity; UACR: urinary albumin:creatinine ratio; CI, confidence interval. *P* <0.05 highlighted in bold.

**Table S5: Longitudinal associations between progression of PWV and baseline and follow-up risk factors, including SBP instead of MAP, using multivariable linear regression analyses**

|  | **Controls** | | | **CKD** | | |  |
| --- | --- | --- | --- | --- | --- | --- | --- |
|  | **β** | **95% CI** | ***P*** | **β** | **95% CI** | ***P*** | |
| **Baseline factors** |  |  |  |  |  |  | |
| Age_B_ (years) | 0.63 | 0.22 – 1.04 | **0.004** | 0.15 | -0.06 – 0.36 | 0.155 | |
| Sex | -0.07 | -0.35 – 0.22 | 0.633 | -0.07 | -0.25– 0.11 | 0.425 | |
| Ethnicity (non-white) | -0.14 | -0.44 – 0.16 | 0.354 | 0.08 | -0.08 – 0.24 | 0.336 | |
| BMIz_B_ | -0.20 | -0.55 – 0.15 | 0.261 | -0.20 | -0.37 – -0.03 | **0.019** | |
| SBP_B_ (mmHg) | -0.06 | -0.52 – 0.40 | 0.788 | 0.22 | 0.01 – 0.44 | **0.045** | |
| HR_B_ (bpm) | 0.36 | -0.03 – 0.70 | **0.034** | 0.04 | -0.16 – 0.23 | 0.711 | |
| PWV_B_ (m/s) | -0.55 | -0.95 - -0.16 | **0.007** | -0.60 | -0.79 – -0.43 | **<0.001** | |
| Antihypertensives (y/n) | - | - | - | 0.09 | -0.17 – 0.35 | 0.494 | |
| eGFR_B_ (ml/min/1.73m^2^/year) | - | - | - | -0.09 | -0.25 – 0.08 | 0.307 | |
|  |  |  |  |  |  |  | |
| **Follow-up measures** |  |  |  |  |  |  | |
| BMIz_AP_ | 0.08 | -0.22 – 0.38 | 0.601 | -0.08 | -0.28 – 0.10 | 0.352 | |
| SBP_AP_ (mmHg) | 0.14 | -0.28 – 0.56 | 0.504 | 0.48 | 0.27 – 0.70 | **<0.001** | |
| HR_AP_ (bpm) | 0.34 | -0.03 – 0.70 | 0.068 | 0.00 | -0.19 – 0.19 | 0.999 | |
| Antihypertensives (y/n) | - | - | - | -0.14 | -0.41 – 0.12 | 0.288 | |
| eGFR_AP_ (ml/min/1.73m^2^/year) | - | - | - | 0.06 | -0.10 – 0.22 | 0.456 | |

Multivariable analysis was performed using the enter method. β = standardised regression coefficient. BMIz: body mass index *z*-score at baseline, BMIz_AP:_ annual progression in body mass index z-score; eGFR_B_: estimated glomerular filtration rate at baseline, eGFR_AP_: annual progression in estimated glomerular filtration rate; HR_B_: heart rate at baseline; HR_AP_: annual progression in heart rate; SBP_B:_ mean systolic blood pressure at baseline; SBP_AP_: annual progression in systolic blood pressure; PWV: pulse wave velocity; CI, confidence interval. *P* <0.05 highlighted in bold.

**Table S6: Longitudinal associations between progression of PWV and baseline and follow-up risk factors, including DBP instead of MAP, using multivariable linear regression analyses.**

|  | **Controls** | | | **CKD** | | |  |
| --- | --- | --- | --- | --- | --- | --- | --- |
|  | **β** | **95% CI** | ***P*** | **β** | **95% CI** | ***P*** | |
| **Baseline factors** |  |  |  |  |  |  | |
| Age_B_ (years) | 0.48 | 0.14 – 0.81 | **0.006** | 0.14 | -0.07 – 0.33 | 0.185 | |
| Sex | 0.03 | -0.22 – 0.28 | 0.819 | -0.08 | -0.25– 0.09 | 0.366 | |
| Ethnicity (non-white) | -0.13 | -0.39 – 0.13 | 0.324 | 0.03 | -0.13 – 0.19 | 0.715 | |
| BMIz_B_ | -0.20 | -0.50 – 0.10 | 0.191 | -0.15 | -0.31 – 0.00 | 0.060 | |
| DBP_B_ (mmHg) | 0.25 | -0.08 – 0.58 | 0.130 | 0.25 | 0.06 – 0.44 | **0.012** | |
| HR_B_ (bpm) | 0.24 | -0.05 – 0.53 | 0.097 | 0.04 | -0.15 – 0.23 | 0.674 | |
| PWV_B_ (m/s) | -0.64 | -0.99 - -0.29 | **<0.001** | -0.59 | -0.76 – -0.41 | **<0.001** | |
| Antihypertensives (y/n) | - | - | - | 0.16 | -0.09 – 0.41 | 0.211 | |
| eGFR_B_ (ml/min/1.73m^2^/year) | - | - | - | -0.05 | -0.21 – 0.11 | 0.518 | |
|  |  |  |  |  |  |  | |
| **Follow-up measures** |  |  |  |  |  |  | |
| BMIz_AP_ | 0.02 | -0.24 – 0.29 | 0.877 | -0.05 | -0.24 – 0.12 | 0.526 | |
| DBP_AP_ (mmHg) | 0.49 | 0.16 – 0.82 | **0.004** | 0.48 | 0.30 – 0.70 | **<0.001** | |
| HR_AP_ (bpm) | 0.19 | -0.07 – 0.45 | 0.148 | 0.09 | -0.08 – 0.27 | 0.299 | |
| Antihypertensives (y/n) | - | - | - | -0.20 | -0.46 – 0.06 | 0.133 | |
| eGFR_AP_ (ml/min/1.73m^2^/year) | - | - | - | 0.10 | -0.06 – 0.26 | 0.200 | |

Multivariable analysis was performed using the enter method. β = standardised regression coefficient. BMIz: body mass index *z*-score at baseline, BMIz_AP:_ annual progression in body mass index z-score; eGFR_B_: estimated glomerular filtration rate at baseline, eGFR_AP_: annual progression in estimated glomerular filtration rate; HR_B_: heart rate at baseline; HR_AP_: annual progression in heart rate; DBP_B:_ diastolic blood pressure at baseline; DBP_AP_: annual progression in diastolic blood pressure; PWV: pulse wave velocity; CI, confidence interval. *P* <0.05 highlighted in bold.
