# Supplementary material for: Arterial stiffening in children with early chronic kidney disease is associated with blood pressure but not decline in kidney function: a longitudinal study from the HOT-KID cohort
Source: Pediatr Nephrol. 2026 Jan 26;41(7):2099–108. doi: 10.1007/s00467-026-07157-1 (PMC13197352; doi:10.1007/s00467-026-07157-1)
Supplement: Supplementary file 2 — Graphical abstract (264 KB PPTX) [file 467_2026_7157_MOESM2_ESM.pptx]

## Slide 1
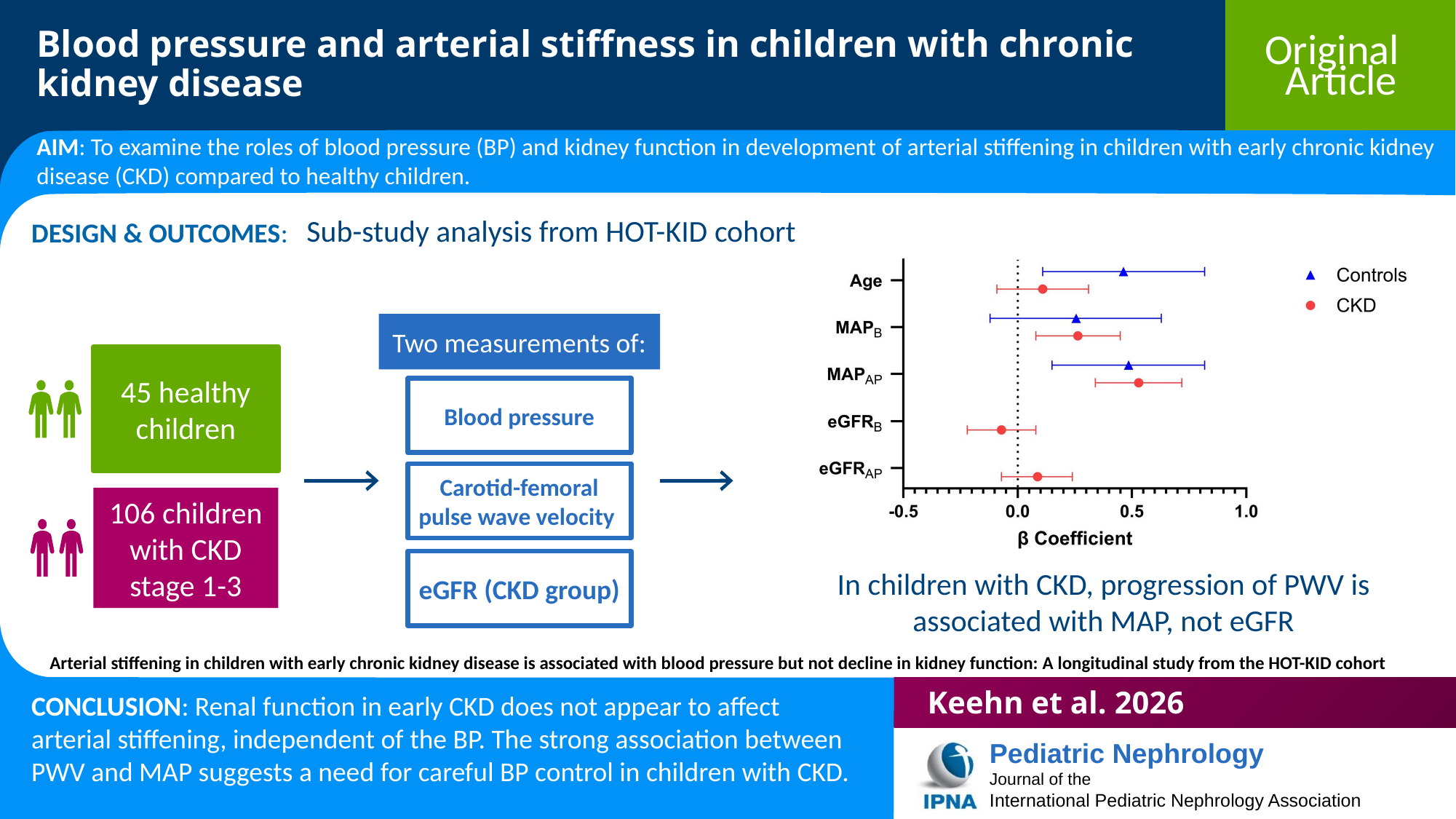

Blood pressure and arterial stiffness in children with chronic kidney disease
AIM: To examine the roles of blood pressure (BP) and kidney function in development of arterial stiffening in children with early chronic kidney disease (CKD) compared to healthy children.
Sub-study analysis from HOT-KID cohort
DESIGN & OUTCOMES:
Two measurements of:
45 healthy children
Blood pressure
Carotid-femoral pulse wave velocity
106 children with CKD stage 1-3
eGFR (CKD group)
In children with CKD, progression of PWV is associated with MAP, not eGFR
Arterial stiffening in children with early chronic kidney disease is associated with blood pressure but not decline in kidney function: A longitudinal study from the HOT-KID cohort
Keehn et al. 2026
CONCLUSION: Renal function in early CKD does not appear to affect arterial stiffening, independent of the BP. The strong association between PWV and MAP suggests a need for careful BP control in children with CKD.
